# Current and Future Perspectives in Mohs Micrographic Surgery for Non-Melanoma Skin Cancers: A Narrative Review

**DOI:** 10.3390/jcm15124754

**Published:** 2026-06-18

**Authors:** A. Paradisi, F. Brunetti, G. M. Jeha, S. N. Tolkachjov

**Affiliations:** 1 Dermatologia, Dipartimento di Medicina e Chirurgia Traslazionale, Università Cattolica del Sacro Cuore, 00168 Rome, Italy; francesco.brunetti@guest.policlinicogemelli.it; 2 UOC di Dermatologia, Dipartimento di Scienze Mediche e Chirurgiche, Fondazione Policlinico Universitario A. Gemelli-IRCCS, 00168 Rome, Italy; 3 Epiphany Dermatology, Dallas, TX 75390, USA; gmjeha@gmail.com (G.M.J.); stan.tolkachjov@gmail.com (S.N.T.); 4 Division of Dermatology, Baylor University Medical Center, Dallas, TX 75390, USA; 5 Department of Dermatology, University of Texas at Southwestern, Dallas, TX 75390, USA; 6 College of Medicine, Texas A&M University, Dallas, TX 75390, USA

**Keywords:** basal cell carcinoma, cutaneous squamous cell carcinoma, Mohs micrographic surgery, presurgical margin demarcation, BCC, SCC, MMS, NMSC

## Abstract

Mohs micrographic surgery (MMS) is a highly specialized skin cancer procedure that combines complete microscopic margin assessment with maximal preservation of uninvolved tissue. The technique is based on staged excision of the tumor with systematic horizontal sectioning and real-time examination of the entire peripheral and deep surgical margins, allowing further tissue removal only in areas where residual tumor is identified. Its unique strength lies in the ability to detect subclinical tumor extensions that may be missed by conventional excision and standard vertical sectioning, thereby improving local control while minimizing unnecessary tissue sacrifice. Since its introduction in the 1930s by Frederic E. Mohs, the technique has evolved into a cornerstone of modern dermato-oncology, particularly for tumors arising in anatomically critical areas, recurrent neoplasms, and histologically aggressive malignancies. MMS is now widely regarded as the treatment of choice for high-risk basal cell carcinoma and cutaneous squamous cell carcinoma because of its superior cure rates and tissue-sparing approach. Beyond its oncologic advantages, MMS allows precise clinicopathologic correlation and immediate reconstruction tailored to the final defect, contributing to favorable functional and cosmetic outcomes. As experience with the technique has expanded, so too has interest in adjunctive tools for preoperative tumor delineation and margin control, further refining patient selection and surgical accuracy. Overall, MMS represents an essential advance over conventional excision for selected cutaneous malignancies, offering an optimal balance between radical tumor clearance and preservation of normal tissue.

## 1. Introduction

Mohs micrographic surgery (MMS) is a specialized skin cancer surgery characterized by complete histopathological analysis of lateral and deep margins of the tumor through horizontal histological sections, which ensures a low recurrence rate while maximizing healthy tissue preservation [1].

Non-melanoma skin cancer (NMSC) represents a major and increasing public health burden worldwide: according to recent GBD 2021 analyses, the age-standardized incidence rate increased from 45.0 to 74.1 per 100,000 between 1990 and 2021, while the age-standardized prevalence rose from 18.4 to 32.5 per 100,000; moreover, the burden increases with age and is higher in men [2,3].

**Figure 1:**
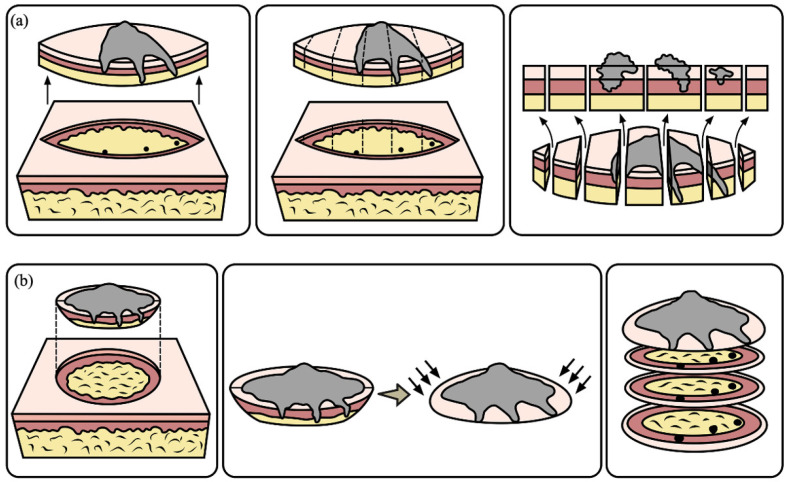
“Bread-loaf” vertical histological section in standard surgical excision (**a**). Horizontal histological sections in Mohs Micrographic Surgery (**b**). Figure adapted and modified from [1].

Most NMSC can be effectively treated with conventional surgery, using appropriate surgical margins for each type of cancer, achieving a low rate of local recurrence and acceptable esthetic results. Other conventional treatment options include radiotherapy, cryosurgery, topical therapies, and photodynamic therapy, although these approaches may provide less complete margin control than MMS, particularly in high-risk tumors. Nevertheless, certain subtypes of cutaneous tumors—because of their histological features, challenging anatomical locations, or clinical history (such as recurrent lesions)—have small extensions or “roots” that can be missed when excised in a “bread-loaf” fashion (Figure 1), leading to higher rates of local recurrence after conventional surgery or would otherwise result in poor cosmetic outcomes. Nowadays, in these cases, MMS represents the treatment of choice [4,5].

## 2. History

First described by Dr. Frederic E. Mohs in the 1930s, it was originally named “chemosurgery” because it involved applying a 20% zinc chloride paste in vivo to fix the tumor before excision. Despite its initial success, this procedure presented several drawbacks. It was painful, labor-intensive and time-consuming because the zinc chloride paste, a strong chemical irritant, required overnight fixation, delaying reconstruction for several weeks [6]. In 1953, while filming a demonstration of the chemosurgery technique on the eyelid, Dr. Mohs preferred fresh-frozen tissue fixation instead of the zinc chloride fixative to speed the filming of the procedure, marking the start of the modern form of MMS [7]. Nowadays, MMS has progressively gained global acceptance and is now regarded as the standard of care for numerous common and rare cutaneous tumors.

## 3. Surgical Technique

**Figure 2:**
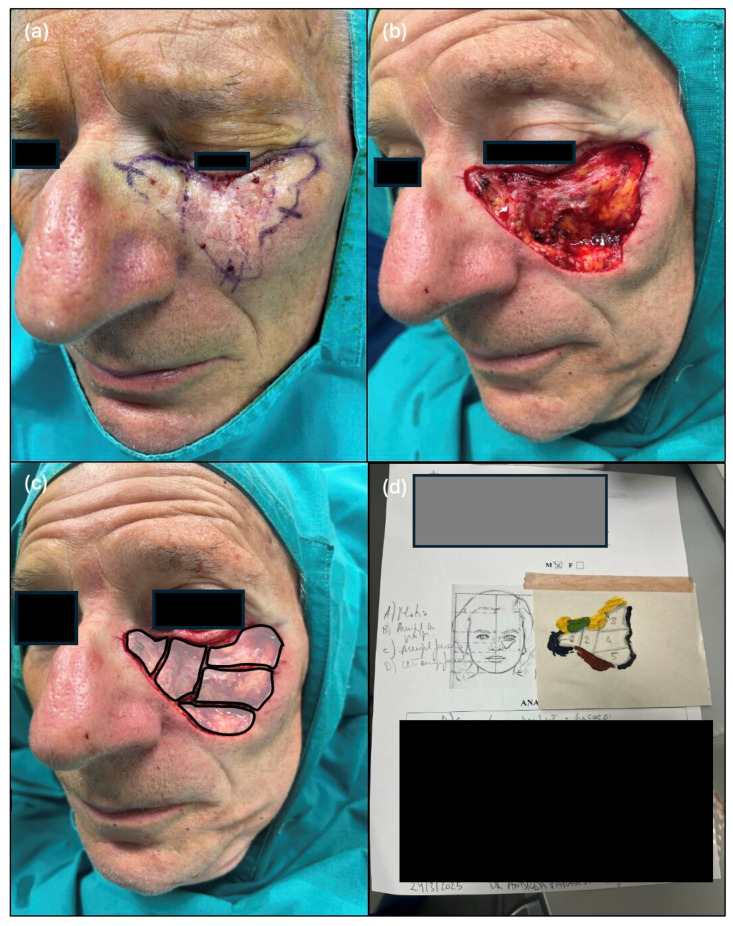
Recurrent basal cell carcinoma of the lower left eyelid in a 73-year-old male patient. After clinical and dermoscopic demarcation of the surgical margin of the tumor, the specimen is orientated with a small vertical incision at the 3, 6 and 12 o’clock positions (**a**). The lesion is then excised with the scalpel held at a 45° angle (**b**). Following excision, the specimen (4 × 3.5 × 0.5 cm) is divided into five pieces (**c**) and subsequentially inked with different colors, one for each lateral margin and one for the deep margin (**d**).

The first crucial step in MMS is the clinical demarcation of the lesion using a dermographic pen, aided by dermoscopic examination, generally maintaining lateral margins of approximately 1–2 mm [8]. This is followed by disinfection of the surgical field and administration of local anesthesia, typically with an anesthetic solution containing adrenaline to enhance intraoperative hemostasis. The lesion is then “debulked” with a curette or scalpel to remove the bulk of the tumor and better delineate its effective size. Prior to excision, the specimen is oriented by making small vertical reference incisions with the scalpel at the 12, 3, and 6 o’clock positions. The excision is then performed with the scalpel held at a 45° angle, which facilitates flattening of the specimen during histologic processing and ensures that the lateral and deep margins lie on the same plane during fixation. Once the tissue has been removed, meticulous hemostasis is achieved using electrocoagulation, ultrasonic coagulation, or vessel ligation [1] (Figure 2a,b).

## 4. Histologic Preparation of the Specimen

A critical step in the preparation of the histologic specimen is the creation of a two-dimensional map of the excised tissue, oriented according to the surgical removal, which allows precise correlation during microscopic evaluation. The specimen is then subjected to a flattening process so that the lateral and deep margins lie on the same cutting plane. This can be facilitated by making small peripheral incisions along the lateral edges. In the case of large lesions, the specimen may be sectioned into multiple parts, typically quadrants, to ensure proper handling and orientation. Subsequently, the tissue is inked with different colors—one for the deep margin and one or more (depending on specimen size) for the lateral margins. The same color pattern is then transferred onto the two-dimensional map to maintain accurate spatial correspondence (Figure 2c,d).

**Figure 3:**
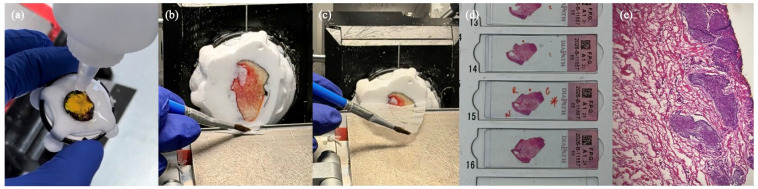
The specimen is then frozen upside-down, embedded in a specific freezing medium (**a**), and multiple horizontal sections are obtained using a cryostat (**b**,**c**). The specimen is then processed, and the resulting histological sections are stained with toluidine blue (**d**) for subsequent visualization under light microscopy (**e**).

The specimen is then frozen and mounted upside down on a chuck, with the dermal surface facing upward, and embedded in a specific freezing medium. Once frozen, serial sections are obtained using a cryostat and are usually stained with hematoxylin and eosin, or alternatively toluidine blue, before microscopic examination by the Mohs surgeon (Figure 3a–d).

**Figure 4:**
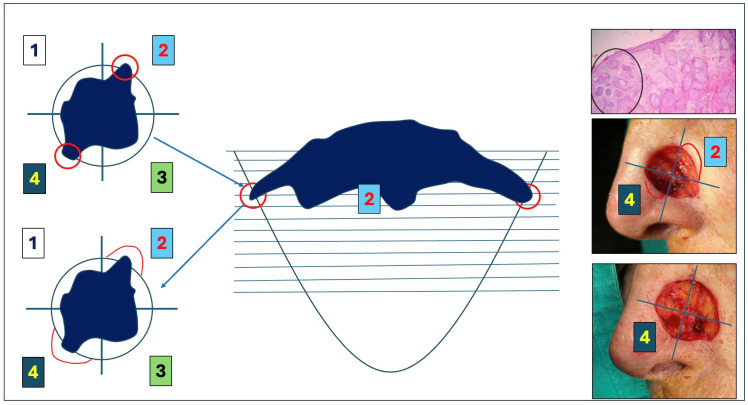
When a tumor is still identified at the peripheral or deep margin on microscopic examination, an additional narrow layer of tissue (approximately 1–2 mm) is removed specifically from the involved area. The specimen is then reoriented, processed and examined under light microscopy. The process is repeated until histologic confirmation of tumor-free margins is obtained.

If residual tumor is identified at either the lateral or deep margins, the surgeon excises an additional 1–2 mm of tissue in the corresponding area, and the entire process is repeated until complete clearance is achieved. After histologic confirmation of tumor-free margins, reconstruction is performed either by direct closure or, when necessary, with local flaps, skin grafts, or dermal substitutes (Figure 4).

## 5. Preoperative Consideration

### 5.1. Selecting the Right Local Anesthetic for MMS

Lidocaine remains the first-line local anesthetic due to its rapid onset (<1 min),th favorable duration of action, and excellent safety profile in MMS [9]; with studies demonstrating that both 0.25% and 0.5% concentrations provide equivalent analgesia and patient satisfaction, while lower concentrations may reduce toxicity risk [10]. Safety data from large patient cohorts indicate that lidocaine toxicity is exceedingly rare in dermatologic surgery, as the volumes typically used remain far below toxic esholds, and adverse effects are generally mild and transient and include circumoral numbness, facial tingling and metallic taste [11]. In a review of local anesthetic systemic toxicity cases from 2010 to 2014, only 2 of 67 cases occurred in dermatologist-performed procedures, both after topical lidocaine/prilocaine application; moreover, only five dermatologist-associated complications were reported in mandatory office-based surgery datasets collected over 16 years, and in a prospective Mohs study using 48 mL of 1% lidocaine with 1:100,000 epinephrine, the highest recorded serum lidocaine level was 0.3 µg/mL [12].

The addition of epinephrine (typically 1:100,000 or 1:200,000 ratio) is standard practice to prolong anesthesia and minimize bleeding, with no significant difference in patient comfort between these two formulations [13]. For longer procedures such as MMS, the use of adjunctive anesthetic such as 0.5% bupivacaine with epinephrine has been shown to prolong anesthesia for at least the interval between Mohs stages: in a randomized trial, the mean time from injection to testing was 76 min, and no patient receiving adjunctive 0.5% bupivacaine with 1:200,000 epinephrine had a positive pain response, compared with 7 of 25 (28%) patients receiving lidocaine alone [14]. Recently, liposomal bupivacaine seems to offer additional benefits by reducing postoperative pain and opioid requirements, although larger studies are still needed to confirm its role in dermatologic surgery [15]. Pain from the administration of a local anesthetic injection remains a common concern. Evidence supports several strategies to minimize injection discomfort–such as buffering with sodium bicarbonate solution, warming the anesthetic, or using skin vibration–as well as providing anticipatory guidance to help manage patient anxiety [9,16].

### 5.2. Discontinuation of Anticoagulant and/or Anti-Platelet Therapy Before MMS

For perioperative management of anticoagulants, the AAD guideline recommends that oral anticoagulant, antithrombotic, and antiplatelet medications are generally continued during dermatologic surgery, as the risk of thromboembolic events from discontinuation outweighs the risk of mild bleeding complications, which are typically manageable and rarely result in serious adverse outcomes [17]. If anticoagulant interruption is considered necessary due to higher bleeding risk (i.e., extensive excision, necessity of wide flap or graft), warfarin should be stopped 5 days prior to achieve an INR < 1.5, and direct oral anticoagulants (apixaban, rivaroxaban, edoxaban) should be stopped 1–2 days before surgery [18,19].

### 5.3. Prophylactic Antibiotics

Routine prophylactic antibiotics are not recommended for most patients undergoing MMS, as surgical site infection rates are low and current evidence does not demonstrate a significant reduction in infection risk with antibiotic use in standard surgery [20,21]. The AAD guidelines specifically advise against routine perioperative systemic antibiotics for adult patients undergoing dermatologic surgery, including MMS [17].

Prophylactic antibiotics may be appropriate for selected high-risk patients or procedures, including those with immunosuppression, diabetes, significant inflammatory skin disease, flap or graft repairs (particularly on the nose or ear), surgery on the lower extremities, or reconstructions performed in a facility-based rather than office setting [21]. According to the APDSAS 2025 advisory statement [22], antibiotic prophylaxis should be given preoperatively, ideally 30–60 min before surgery. For prevention of surgical site infection in nonmucosal high-risk cases, cephalexin 2 g orally is recommended; in rare patients with contraindications to cephalosporins, trimethoprim-sulfamethoxazole 160/800 mg orally or doxycycline 100 mg orally may be considered as single preoperative doses. For prevention of infective endocarditis or prosthetic joint infection, prophylaxis is indicated only when surgery involves the oral mucosa or clinically infected skin in at-risk patients; in these settings, amoxicillin 2 g orally is recommended for oral mucosal procedures, whereas cephalexin 2 g orally may be appropriate for infected skin. In rare patients with contraindications to amoxicillin, levofloxacin 500 mg plus clindamycin 600 mg orally may be used as a single preoperative dose.

Nonetheless, recent evidence—including a large retrospective analysis of lower-extremity Mohs cases—demonstrates no meaningful reduction in infection rates with prophylactic antibiotics, even for complex closures. Moreover, unnecessary antibiotic use contributes to adverse effects and antimicrobial resistance [23].

## 6. Different Surgical Techniques in MMS

Several adaptations of standard MMS have been developed to address specific cutaneous tumors and locations, such as the “*Tubingen technique*”, “*muffin technique*” and “*slow-Mohs*”.

The “*Tubingen technique*” or “*Tubingen torte*” or “*cake technique*” uses 3D-paraffin histology to achieve precise margin control. After tumor excision, the specimen is processed in a way that allows for precise evaluation of all peripheral and deep margins by separating the first from the latter for different histopathological examinations, enabling accurate mapping of residual tumor and maximal preservation of healthy tissue. This approach is particularly favorable for large tumors with indistinct margins, such as dermatofibrosarcoma protuberans (DFSP), and has demonstrated significant tissue-sparing benefits and low recurrence rates in comparative studies with wide local excision (WLE) [24,25].

The “*muffin technique*” excises the lesion with a narrow margin and processes the entire specimen in paraffin for postoperative margin assessment, providing full margin control with recurrence rates comparable to standard MMS [26].

“*Slow-Mohs*” uses formalin-fixed, paraffin-embedded sections rather than frozen tissue, allowing for immunohistochemical staining when needed (i.e., lentigo maligna, DFSP) and offering high cure rates, with the main drawback being delayed histologic evaluation [27].

## 7. Limitations of MMS

Despite its established efficacy, MMS carries several important limitations that warrant discussion. Its higher cost relative to conventional excision and restricted availability outside specialized academic centers limit access for many patients. The technique requires dedicated laboratory infrastructure, trained support staff, and a surgeon with subspecialty expertise, creating a meaningful learning curve and constraining dissemination to community settings. Intraoperative frozen-section processing, while enabling same-day margin evaluation, is subject to artifact and tissue distortion that may occasionally compromise histologic interpretation. Furthermore, the superior cure rates of MMS are most evident for high-risk tumors; for low-risk lesions in non-critical anatomic locations, conventional excision with standard margins achieves comparable outcomes, and routine use of MMS in this setting may constitute overtreatment [28]. Careful patient selection guided by established appropriate use criteria remains essential to ensure that MMS is applied where its benefits are most clearly justified.

## 8. Mohs Micrographic Surgery for Basal Cell Carcinoma

### 8.1. Epidemiology

Basal cell carcinoma (BCC) is the most common skin tumor and the most common type of cancer in humans; it accounts for 75% of all NMSC. Estimating the true incidence of BCC is challenging because many cases are not recorded in national cancer registries, and some subtypes do not require histopathological confirmation, as they can be effectively managed with topical treatments or destructive techniques [29]. Nonetheless, the incidence continues to rise globally, with the highest rates observed in populations with lighter skin phototypes and in regions such as North America, Australia, and Western Europe [30]. BCC typically presents on sun-exposed areas, especially the head and neck, and is characterized by indolent growth and a very low risk of metastasis but can cause significant local morbidity through tissue destruction [31].

### 8.2. Staging

**Figure 5:**
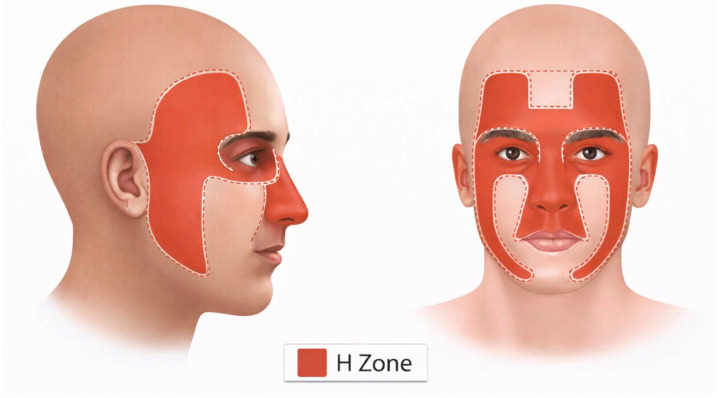
H zone of the face.

Most cases of BCC are diagnosed at an early stage and can be effectively treated with surgery or topical and destructive therapies. However, certain BCC subtypes, depending on tumor or patient characteristics, may not be amenable to conventional surgery (Figure 5).

According to the European Association of Dermato-Oncology (EADO) guidelines, these BCCs are considered difficult to treat when factors such as tumor size or location, indistinct margins, morpheaform histology, multiple recurrences, prior radiotherapy, or patient-related surgical limitations compromise functional or esthetic outcomes [5].

On the other hand, American guidelines stratify BCC in low-risk and high-risk for local recurrence according to the National Comprehensive Cancer Network (NCCN) based on clinical and pathological parameters (i.e., location, borders, primary vs. recurrent, immunosuppression, prior radiotherapy, growth pattern and perineural involvement) [32].

### 8.3. Indications for Mohs Micrographic Surgery for Basal Cell Carcinoma

In the United States, indications to MMS are based on the Appropriate Use Criteria (AUC).

**Figure 6:**
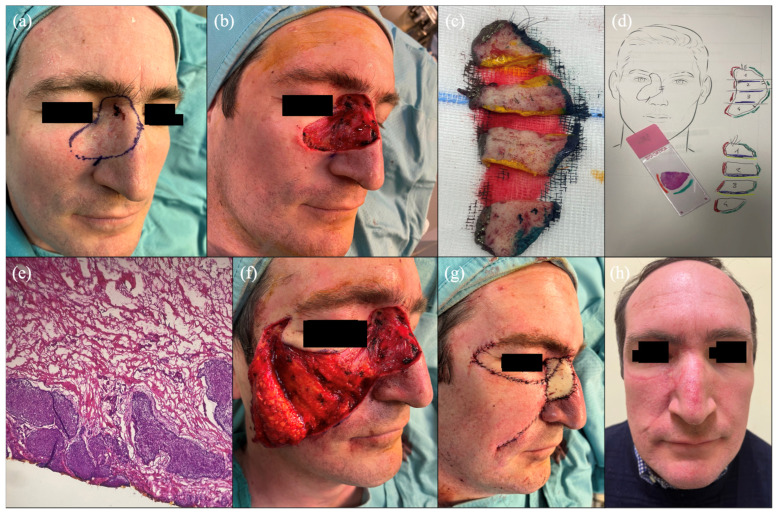
Recurrent basal cell carcinoma (BCC) involving multiple anatomical subunits of the face—inner cantus of the eye, lateral wall of the nose and infraorbital zone—in a 47-year-old male patient (**a**). Post-excisional defect following the first stage of Mohs Micrographic Surgery (MMS) (**b**). The surgical specimen was divided into four sections, each marked with different colors to identify the corresponding lateral and deep margins (**c**). The same color-coding system was transferred onto a two-dimensional surgical map to ensure precise spatial orientation and margin correlation (**d**). Histologic image showing multiple basaloid tumor islands within the dermis, consistent with BCC (**e**). According to facial subunits reconstruction principles, the defect was reconstructed with a right cheek advancement flap; the inner cantus and the lateral wall of the nose were repaired with a graft; the former using the upper orbicularis Burow’s triangle and the latter with the opposite semilunar variant of the Burow triangle from the melolabial fold (**f**,**g**). Final clinical outcome at 3 months follow-up after MMS (**h**).

The AUC was jointly developed by the American Academy of Dermatology (AAD), American College of Mohs Surgery (ACMS), American Society for Dermatologic Surgery Association (ASDSA), and American Society for Mohs Surgery (ASMS), integrating evidence-based data with expert consensus. This process led to the evaluation and rating of hundreds of clinical scenarios to help clinicians identify those in which MMS provides the best clinical outcomes. Indications for MMS in BCC are primarily determined by clinical and histopathologic risk factors related to recurrence, subclinical spread, and the need for maximal tissue preservation. Clinically, high-risk features for which MMS is indicated are localization within the “H-area”, which includes the “mask area of the face”—specifically the eyelids, nose, lips, eyebrows, chin, temples, ears and preauricular skin—as well as involvement of functionally or cosmetically sensitive sites such as the hands, feet, ankles, nail units, nipple/areola complex or genitalia (Figure 6).

Tumors localized within the “M-area”, which includes the cheeks, forehead, neck, jawline, scalp and pretibial surface, are also eligible for MMS, with a high “appropriate use score”, except for superficial BCC smaller than 0.6 cm arising in healthy patients.

By contrast, on the “L-area” of the skin, which includes the trunk and extremities (excluding the pretibial surface, hands, feet, nail unit and ankles), indications for MMS are: recurrent aggressive and nodular BCC; primary aggressive BCC > 0.5 cm; primary nodular BCC (>2 cm in healthy patients and >1 cm in immunocompromised patients), whereas primary superficial BCC did not achieve consensus for indication to MMS.

Additional high-risk factors, for which MMS should be considered, are poorly defined tumor borders, prior radiation therapy to the area, early onset (<40 years), and the presence of genetic syndromes such as Gorlin syndrome or xeroderma pigmentosum. From a histopathologic perspective, aggressive subtypes include morpheaform, infiltrative, micronodular and basosquamous variants along with perineural invasion [1,32,33] (Table 1).

**Figure 7:**
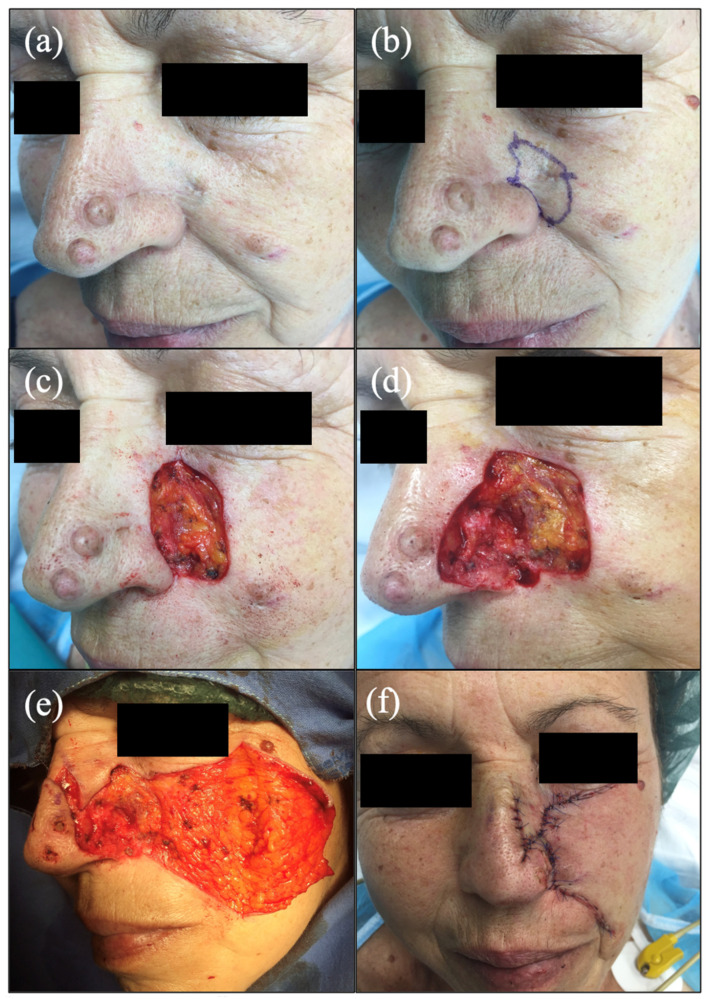
Primary basal cell carcinoma with ill-defined borders, apparently involving the left cheek, in a 65-year-old female patient (**a**). The clinical margin of the tumor was demarcated with the help of dermoscopy (**b**). Post-excisional defect following the first stage of MMS (**c**). Final defect following five stages of MMS. The tumor had involved different anatomical subunits of the face: left cheek, nasal ala and sidewall (**d**). According to facial subunits reconstruction principles, the defect was reconstructed with a left cheek advancement flap; the sidewall and ala of the nose were repaired with a transposition flap from the dorsum of the nose (**e**). The patient immediately after surgery (**f**).

In Europe, with the exclusion of Germany, Switzerland and the Netherlands, where MMS is frequently performed, this technique is not as widely used as in the United States because of the high cost of the procedure and is offered only in a few specialized centers. According to EADO guidelines, MMS shall be offered in high-risk BCC, specifically in recurrent tumors, aggressive subtypes, locations in critical anatomical sites and poorly defined margins [5] (Figure 7).

## 9. Preoperative Evaluation in MMS

Pre-operative margin demarcation of BCC before performing MMS plays a key role in achieving histopathological clearance within the first stage of surgery, preserving healthy tissue removal.

Various techniques have been investigated for surgical margin delineation, including dermoscopy, reflectance confocal microscopy (RCM), optical coherence tomography (OCT), and line-field confocal optical coherence tomography (LC-OCT) [34].

### 9.1. Dermoscopy

Dermoscopy is a non-invasive imaging technique widely employed for the diagnosis of skin cancer; many studies have investigated the effectiveness of its use in tumor margin delineation for MMS [35,36,37,38].

While several studies have reported that dermoscopy significantly reduces the number of MMS stages, final defect size, and recurrence rates [35,37], other investigations [38,39,40] have not demonstrated a meaningful reduction in the number of MMS stages when dermoscopy is employed.

Given its low cost and noninvasive nature, it is widely employed to refine surgical margin delineation after initial clinical demarcation.

### 9.2. Reflectance Confocal Microscopy (RCM)

RCM is a non-invasive imaging technique that allows real-time, in vivo visualization of the epidermis and superficial dermis at near-histologic resolution, making it particularly suitable for preoperative margin assessment in BCC [41,42]. In a prospective study evaluating dermoscopy combined with RCM for lateral margin assessment, RCM showed high specificity (95.2%) and good overall accuracy (79%) for detecting tumor involvement within 2 mm of the surgical border [43].

### 9.3. Optical Coherence Tomography (OCT)

OCT is a non-invasive imaging modality that provides real-time, cross-sectional visualization of the epidermis and superficial dermis up to a depth of approximately 1.5–2 mm, allowing in vivo assessment of tumor architecture before surgery. When applied for presurgical margin assessment, OCT has shown good correlation with histopathology, correctly identifying lateral margins in 84% of non-melanoma skin cancers in a clinical study [44]. In a pilot study evaluating OCT-guided margin refinement prior to MMS, OCT margins were consistently within the final defect in single-stage cases and predicted subclinical extension in lesions requiring additional stages [45]. These findings suggest that OCT can improve the accuracy of preoperative tumor delineation, potentially reducing the number of surgical stages and sparing healthy tissue without compromising oncologic safety.

### 9.4. Line-Field Confocal Optical Coherence Tomography (LC-OCT)

Line-field confocal optical coherence tomography (LC-OCT) is a novel, non-invasive, real-time, in vivo imaging technique that integrates horizontal and vertical imaging with three-dimensional reconstructions at single-cell resolution, providing optical images with OCT-like penetration depth (up to approximately 500 µm) and reflectance confocal microscopy (RCM)-like resolution (lateral resolution of ~1.3 µm and axial resolution of ~1.1 µm). Nowadays, there is growing interest in the use of LC-OCT as a non-invasive technique for preoperative delineation of surgical margins. Following dermoscopic demarcation of the tumor, LC-OCT is employed to achieve cellular-level definition of the lateral margins, particularly in tumors with poorly defined borders: recent studies demonstrated that the use of LC-OCT significantly reduced the number of MMS stages in high-risk BCC [46].

It should be noted that the evidence base for RCM, OCT, and LC-OCT in preoperative margin delineation remains limited and heterogeneous, with most published data derived from small, single-center cohorts and few prospective controlled studies. At this time, these modalities are best considered adjunctive and emerging rather than established standard-of-care tools, and their integration into routine MMS practice should be interpreted with appropriate caution pending larger, prospective validation.

## 10. Future Perspectives

Future developments in preoperative evaluation before MMS are increasingly focused on artificial intelligence (AI). Convolutional neural networks (CNNs) have demonstrated high diagnostic performance in detecting non-melanoma skin cancers on frozen sections and other imaging modalities, supporting their potential role as adjunctive tools for margin control [47]. In addition, AI-based systems are being explored for preoperative tumor mapping and prediction of margin involvement. OCT-based algorithms have shown good accuracy in distinguishing tumor from healthy tissue, with results indicating reliable discrimination between positive and negative margins, potentially reducing the number of surgical stages [48]. Despite these promising results, several challenges remain, including limited external validation, potential algorithmic bias related to non-representative training datasets, data privacy concerns, and the limited interpretability of many deep learning models. At present, AI should be considered a decision-support tool rather than a replacement for surgeon expertise [49].

## 11. MMS Outcomes

MMS has been shown to provide better local disease control and reduced recurrence rates than standard surgical excision (SE) in various randomized controlled trials (RCT) and meta-analysis [28,50,51,52,53,54]. An RCT conducted by Mosterd et al. [51] in the Netherlands between 1999 and 2002 compared MMS and SE in the treatment of primary and recurrent BCC of the face, showing lower recurrences in the group of BCC treated with MMS with a 5-year follow-up. Specifically, 198 pBCC were treated with MMS and 199 primary BCC with SE; among those, 11 recurrences of primary BCC were registered during the 60-month follow-up period, four (2.5%) after MMS and seven (4.1%) after SE. On the other hand, 100 recurrent BCCs were treated with MMS and 102 recurrent BCCs with SE; after the follow-up period of 5 years, 12 recurrences occurred: two (2.4%) in the MMS group and ten (12.1%) in the SE group [51].

Van Loo et al. [28] published in 2014 the result of an RCT on recurrence rate after MMS versus SE with an outstanding 10-year follow-up in pBCC and rBCC. During the 10-year follow-up period, 21 recurrences were registered in the pBCC group (15 after SE and 6 after MMS) with a 10-year cumulative probability of recurrence of 4.4% in the MMS group and 12.2% in the SE group. Moreover, 14 recurrences were registered in the rBCC group (11 after SE and 3 after MMS), with a 10-year cumulative probability of recurrence of 3.9% in the MMS group and 13.5% in the SE group.

More recently, a systematic review and meta-analysis conducted by Lacerda et al. [50] confirmed a lower recurrence rate of BCC and cutaneous squamous cell carcinoma (cSCC) treated with MMS compared to those treated with SE. The meta-analysis evaluated data from seventeen studies: a total of 3050 tumors treated with MMS experienced recurrence in 82 cases, resulting in an overall recurrence rate (ORR) of 3.1%, in contrast with the SE group with 209 recurrences among 3453 tumors and an ORR of 5.3%. Specifically in the BCC subgroup, the incidence rate ratio for recurrence was 0.37, indicating a substantial reduction in recurrence rates when treated with MMS.

It should be noted that conventional excision with adequate surgical margins remains appropriate and effective for low-risk BCC in non-critical anatomic locations, and MMS should not be considered the default approach in this context. For elderly or frail patients in whom surgery carries elevated risk, minimally invasive tissue-sparing alternatives merit consideration. Electrochemotherapy with bleomycin, for example, has demonstrated efficacy for BCC in this population and may serve as a viable option when surgical intervention is not optimal [55].

## 12. Mohs Micrographic Surgery for Cutaneous Squamous Cell Carcinoma

### 12.1. Epidemiology and Clinical Significance

cSCC is the second most common NMSC after BCC [56,57,58]. While cSCC accounts for up to 25% of NMSC cases, it is the leading cause of NMSC death [57,59,60]. The incidence of cSCC is rising across all ages, a phenomenon thought to be due in part to increased surveillance and awareness, aging populations, cumulative ultraviolet exposure, and increased immunosuppression [57,61]. Immunosuppressed patients, including those with solid organ transplants, lymphoid malignancies, or receiving chronic immunomodulatory therapies, have a 40–250 times higher incidence of cSCC compared to the general population [62,63]. This wide range reflects the presence of additional risk factors, including lighter skin type, cumulative ultraviolet exposure, older age at transplantation, and the degree and duration of immunosuppression [64,65]. High-risk human papillomavirus (HPV) types further contribute to carcinogenesis in these patients, leading to anal, genital, cervical, and digital SCC [66]. cSCC occurs more frequently in men, and men also experience nearly threefold higher cSCC-related mortality compared to women [67,68]. cSCC arising in certain anatomic sites, including the ear, lip, genitalia, and within pre-existing scars, are associated with a disproportionately higher risk of local recurrence and disease-specific death [69,70]. Aggressive subclinical extension is particularly common in men, immunosuppressed individuals, and patients with smoking history [70,71].

### 12.2. Prognostic Staging and Risk Factors

The two principal prognostic staging systems currently used for cSCC are the 8th edition of the American Joint Committee on Cancer (AJCC8) and the Brigham and Women’s Hospital (BWH) systems [72]. Both systems improve on earlier classifications and correlate with survival, but AJCC8 shows limited discrimination between T2 and T3, creating a broad 23% subset of head and neck cSCC with substantial risk of metastasis and death—too large a group to uniformly recommend nodal staging or adjuvant therapy [72,73,74]. In contrast, the BWH system identifies a similar number of poor outcomes within only 9% of cases, demonstrating higher specificity (93%) and positive predictive value (30%) for predicting nodal metastasis or disease-specific death compared with AJCC8 [72]. Still, a small subset of BWH T1 tumors with minor risk factors—insufficient to meet major BWH criteria—such as moderate differentiation, 1–2 cm diameter, or subcutaneous fat invasion, may still recur or metastasize, and this risk increases cumulatively with the number of these factors present [75]. Similarly, among BWH T2b tumors, the number of risk factors has been shown to independently predict adverse outcomes [76]. Additional features such as satellitosis (in-transit metastasis), lymphovascular invasion, and ear, temple, or genital location have likewise been independently associated with poorer prognosis [75,77,78].

### 12.3. Indications for Mohs Micrographic Surgery for Squamous Cell Carcinoma

**Figure 8:**
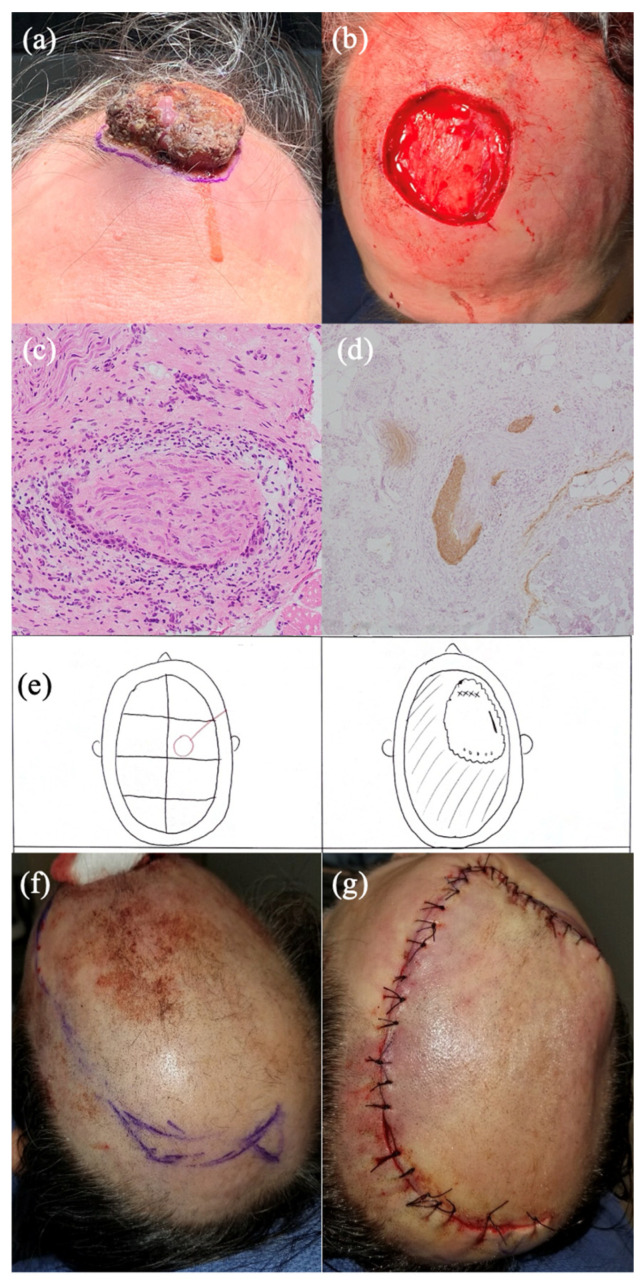
Primary squamous cell carcinoma of the scalp (**a**). A sharp tumor debulk of the clinical nodule was performed and submitted for vertical sectioning to assess tumor differentiation and evaluate for perineural invasion (PNI), followed by curettage and subsequent excision of the residual tumor via standard Mohs technique (**b**). The first Mohs stage was processed as eight tissue blocks; histologic examination demonstrated squamous cell carcinoma with large-caliber PNI in block #4 extending to the level of the subcutaneous fat (**c**,**d**). Clearance was achieved after two stages to the level of the galea, with the second stage consisting of two tissue blocks, followed by the reconstruction of the defect with a large rotational flap (**e**–**g**). The Mohs map is oriented with the patient’s anterior (nose) facing forward for reference; the tumor is not drawn to scale.

Similar to BCC, the indications for MMS in cSCC are generally guided by the Appropriate Use Criteria (AUC), which take into account tumor characteristics, anatomic location, and patient factors to determine when MMS is most appropriate [33]. According to the AUC, Area H (which includes high-risk anatomic regions such as the central face, eyelids, nose, lips, ears, genitalia, hands, and feet) is considered high-risk for cSCC based on location alone, regardless of tumor size, and therefore MMS is almost always appropriate in this context (Figure 8). In contrast, MMS is appropriate in Areas M and L (cheeks, forehead, scalp, neck, trunk, and extremities) for cSCC > 1 cm and >2 cm, respectively, or when high-risk tumor features or specific patient factors are present (Table 2).

### 12.4. Advantages of Mohs Micrographic Surgery for Cutaneous Squamous Cell Carcinoma

The two main advantages of MMS over wide local excision are (1) its complete circumferential peripheral and deep margin assessment, which allows examination of 100% of the true surgical margin, and (2) its tissue-sparing nature. By combining margin control with tissue conservation, MMS achieves the highest cure rates while preserving function and appearance in functionally or cosmetically critical sites such as the eyelids, nose, lips, ears, digits, or genitalia [79,80]. It is especially advantageous for recurrent cSCC, where prior surgery or radiation may obscure margins and compromise orientation, and for poorly differentiated or desmoplastic subtypes, in which tumor infiltration often extends beyond clinical borders [1,81]. In addition, MMS is especially useful in treating cSCC in immunosuppressed patients given the higher-risk nature of cSCC in these populations [82,83]. MMS may also reduce the need for re-excision or complex secondary reconstruction associated with incomplete standard excision and has been reported to be cost-effective for the management of intermediate- and high-risk cSCC [84]. In short, MMS has emerged as the treatment of choice for high-risk or recurrent cSCC, and an essential component of patient care in modern dermatologic oncology.

### 12.5. Outcomes

MMS has demonstrated superior local control and lower recurrence rates compared with wide local excision, particularly for high-risk or recurrent cSCC [70,79,80]. One large cohort study of 579 patients with 672 cSCC found a recurrence rate of 8% after standard excision compared with 3% after MMS, with tumors treated by MMS having a threefold lower adjusted risk of recurrence [85]. In another study involving patients specifically with high-risk cSCC treated with MMS, 2.9% developed local recurrence [70]. More recently, a systematic review comparing outcomes of cSCC treated with MMS versus conventional excision concluded that MMS is generally safer and more effective [80]. Collectively, these data support MMS as the treatment of choice for achieving the highest cure rates and lowest recurrence for cSCC, particularly when high-risk or recurrent, while offering the added benefit of tissue conservation.

## 13. Mohs Micrographic Surgery for Other Rare Non-Melanoma Skin Cancer

MMS is also relevant for selected rare NMSCs beyond BCCs and cSCCs, particularly when tumors show infiltrative growth, subclinical extension, or arise in anatomically critical sites. In DFSP, the characteristic tentacle-like spread into surrounding tissue makes complete margin control especially important; accordingly, slow Mohs/MMS has shown excellent local control, with one comparative series reporting no recurrences after slow Mohs versus 12.5% after wide local excision [27], and a recent meta-analysis suggesting a survival advantage for MMS in recurrent tumors [86]. Eccrine porocarcinoma is another rare adnexal carcinoma in which MMS appears particularly valuable, as historical wide local excision series have reported substantial rates of local recurrence and metastasis, whereas published MMS series have shown no local recurrences or disease-specific deaths during follow-up [87,88]. Sebaceous carcinoma, especially in periocular sites, may likewise benefit from the tissue-sparing nature and complete margin assessment of MMS; in a recent systematic review, MMS was associated with lower local, regional, and distant recurrence rates compared with wide local excision [89]. Although the available evidence is largely retrospective, these data support MMS as an important treatment option for selected rare adnexal and mesenchymal cutaneous malignancies.

## Figures and Tables

**Table 1:** Indications for Mohs micrographic surgery for BCC.

Feature	High-Risk BCC	Low-Risk BCC
Location/size	Area H (any size) Area M (most subtypes) Area L with specific criteria	Area L without high-risk features
Area H involvement	Eyelids, nose, lips, eyebrows, chin, temples, ears, preauricular skin; hands, feet, ankles, nail unit, nipple/areola complex, genitalia	–
Area M involvement	Cheeks, forehead, neck, jawline, scalp, pretibial surface (except superficial BCC < 0.6 cm in healthy patients)	Superficial BCC < 0.6 cm in healthy patients
Area L involvement	Recurrent aggressive or nodular BCC Primary aggressive BCC > 0.5 cm Primary nodular BCC > 2 cm (healthy) Primary nodular BCC > 1 cm (immunocompromised)	Primary superficial BCC
Borders	Poorly defined	Well defined
Primary vs. recurrent	Recurrent	Primary
Site of prior radiation therapy	Yes	No
Age at onset	<40 years	≥40 years
Genetic syndromes	Gorlin syndrome, xeroderma pigmentosum	No
Histologic subtype	Morpheaform, infiltrative, micronodular, basosquamous	Superficial, nodular
Perineural invasion	Present	Absent

**Table 2:** Indications for Mohs micrographic surgery for cSCC.

	High-Risk cSCC	Low-Risk cSCC
Clinical Characteristics		
Location/size	Area H Area M ≥ 1 cm Area L ≥ 2 cm	Area M < 1 cm Area L < 2 cm
Borders	Poorly defined	Well defined
Primary vs. recurrent	Recurrent	Primary
Site of prior radiation therapy or chronic inflammatory process	Yes	No
Rapidly growing tumor	Yes	No
Neurologic symptoms	Yes	No
Pathologic Characteristics		
High-risk subtype	Yes	No
Degree of differentiation	Poorly differentiated	Well or moderately differentiated
Depth	≥2 mm or Clark level IV, V	<2 mm or Clark level I, II, III
Perineural or lymphovascular involvement	Yes	No

## Data Availability

This is a narrative review based on the previously published literature. No new data were created or analyzed in this study. Data sharing is not applicable to this article.
